# Logistics Workers Are a Key Factor for SARS-CoV-2 Spread in Brazilian Small Towns: Case-Control Study

**DOI:** 10.2196/30406

**Published:** 2021-09-01

**Authors:** Breno Bernardes-Souza, Saulo Ricardo Costa Júnior, Carolina Ali Santos, Raimundo Marques Do Nascimento Neto, Fernando De Carvalho Bottega, Daiana Carolina Godoy, Bruno Lourençoni Freitas, Daniela Leite Garcia Silva, Titus Josef Brinker, Raiza Aranha Nascimento, Unaí Tupinambás, Alexandre Barbosa Reis, Wendel Coura-Vital

**Affiliations:** 1 School of Medicine Federal University of Ouro Preto Ouro Preto Brazil; 2 School of Medicine Federal University of Lavras Lavras Brazil; 3 Faculty of Medicine Federal University of Minas Gerais Belo Horizonte Brazil; 4 German Cancer Research Center Heidelberg Germany; 5 School of Medicine Jose do Rosario Vellano University Belo Horizonte Brazil; 6 School of Pharmacy Federal University of Ouro Preto Ouro Preto Brazil; 7 National Institute of Science and Technology in Tropical Diseases Brazilian National Council for Scientific and Technological Development Brasília Brazil

**Keywords:** COVID-19, SARS-CoV-2, logistics workers, risk factors, household infection

## Abstract

**Background:**

Data on how SARS-CoV-2 enters and spreads in a population are essential for guiding public policies.

**Objective:**

This study seeks to understand the transmission dynamics of SARS-CoV-2 in small Brazilian towns during the early phase of the epidemic and to identify core groups that can serve as the initial source of infection as well as factors associated with a higher risk of COVID-19.

**Methods:**

Two population-based seroprevalence studies, one household survey, and a case-control study were conducted in two small towns in southeastern Brazil between May and June 2020. In the population-based studies, 400 people were evaluated in each town; there were 40 homes in the household survey, and 95 cases and 393 controls in the case-control study. SARS-CoV-2 serology testing was performed on participants, and a questionnaire was applied. Prevalence, household secondary infection rate, and factors associated with infection were assessed. Odds ratios (ORs) were calculated by logistic regression. Logistics worker was defined as an individual with an occupation focused on the transportation of people or goods and whose job involves traveling outside the town of residence at least once a week.

**Results:**

Higher seroprevalence of SARS-CoV-2 was observed in the town with a greater proportion of logistics workers. The secondary household infection rate was 49.1% (55/112), and it was observed that in most households (28/40, 70%) the index case was a logistics worker. The case-control study revealed that being a logistics worker (OR 18.0, 95% CI 8.4-38.7) or living with one (OR 6.9, 95% CI 3.3-14.5) increases the risk of infection. In addition, having close contact with a confirmed case (OR 13.4, 95% CI 6.6-27.3) and living with more than four people (OR 2.7, 95% CI 1.1-7.1) were also risk factors.

**Conclusions:**

Our study shows a strong association between logistics workers and the risk of SARS-CoV-2 infection and highlights the key role of these workers in the viral spread in small towns. These findings indicate the need to focus on this population to determine COVID-19 prevention and control strategies, including vaccination and sentinel genomic surveillance.

## Introduction

SARS-CoV-2 emerged in Wuhan, China in December 2019. The virus spread worldwide, resulting in the COVID-19 pandemic [1]. In Brazil, the first case was confirmed on February 25, 2020, and the country gradually became one of the most affected, sustaining an average of more than 40,000 new cases per day and 1000 deaths per day during the second quarter of 2020 [2,3].

As evidence mounted suggesting that a high proportion of individuals infected with SARS-CoV-2 are asymptomatic or oligosymptomatic [4], seroprevalence studies emerged as an important tool not only to see the real extension of the pandemic but also to help understand the dynamics and factors that contribute to viral spread. A national population-based study with samples from 133 large sentinel cities in Brazil conducted from May to June 2020 showed a marked variability in seroprevalence across Brazilian regions, ranging from below 1% in most cities in the south to up to 25% in the Amazon (north) region [5]. Seroprevalence was similar between different ages and sex but was higher among those with low socioeconomic status and among those living in households with greater numbers of people. The study estimated that there were 7 undetected SARS-CoV-2 cases for every detected case in Brazil.

This aforementioned national study included only large Brazilian cities because few cases had been reported in less populous areas at the time. In this context of few reported cases and lack of SARS-CoV-2 research studies in small Brazilian towns, our study aimed to verify the seroprevalence and underreporting of SARS-CoV-2 in these towns, to understand their dynamics of viral transmission, and to identify potential core groups that can serve as an initial source of infection to their general population as well as factors associated with higher risk of infection.

## Methods

### Study Design

Initially, a cross-sectional population-based seroprevalence study was conducted on May 30 and 31, 2020, in the urban area of a small Brazilian town called Nepomuceno (hereby entitled Town 1). Town 1 was chosen by convenience among small towns with no reported COVID-19 cases to verify if SARS-CoV-2 had already spread even in low densely populated Brazilian areas without confirmed COVID-19 cases. The study showed a low seroprevalence, and the identified cases were all related to logistics workers. To confirm the influence of these workers in the spread of the virus in the region, we conducted other studies in another nearby small town called Carmópolis and hereby entitled Town 2.

A similar population-based seroprevalence study was conducted in Town 2 on June 27 and 28, 2020. After the seroprevalence study, a household survey was conducted on June 29 and 30 in all residences of Town 2 that had at least one COVID-19 case confirmed by either our seroprevalence study or by the local health authorities until June 28. During this household survey, all residents were interviewed and serologically tested for COVID-19. After receiving the serological results from a specific household and after evaluating all its individual questionnaires, the interviewer returned to that household to inform them of the results and to conduct a joint interview with all its members aiming to define the index patient and the most likely source of infection to the household and to obtain information on the household’s general COVID-19 prevention behaviors.

**Figure 1:**
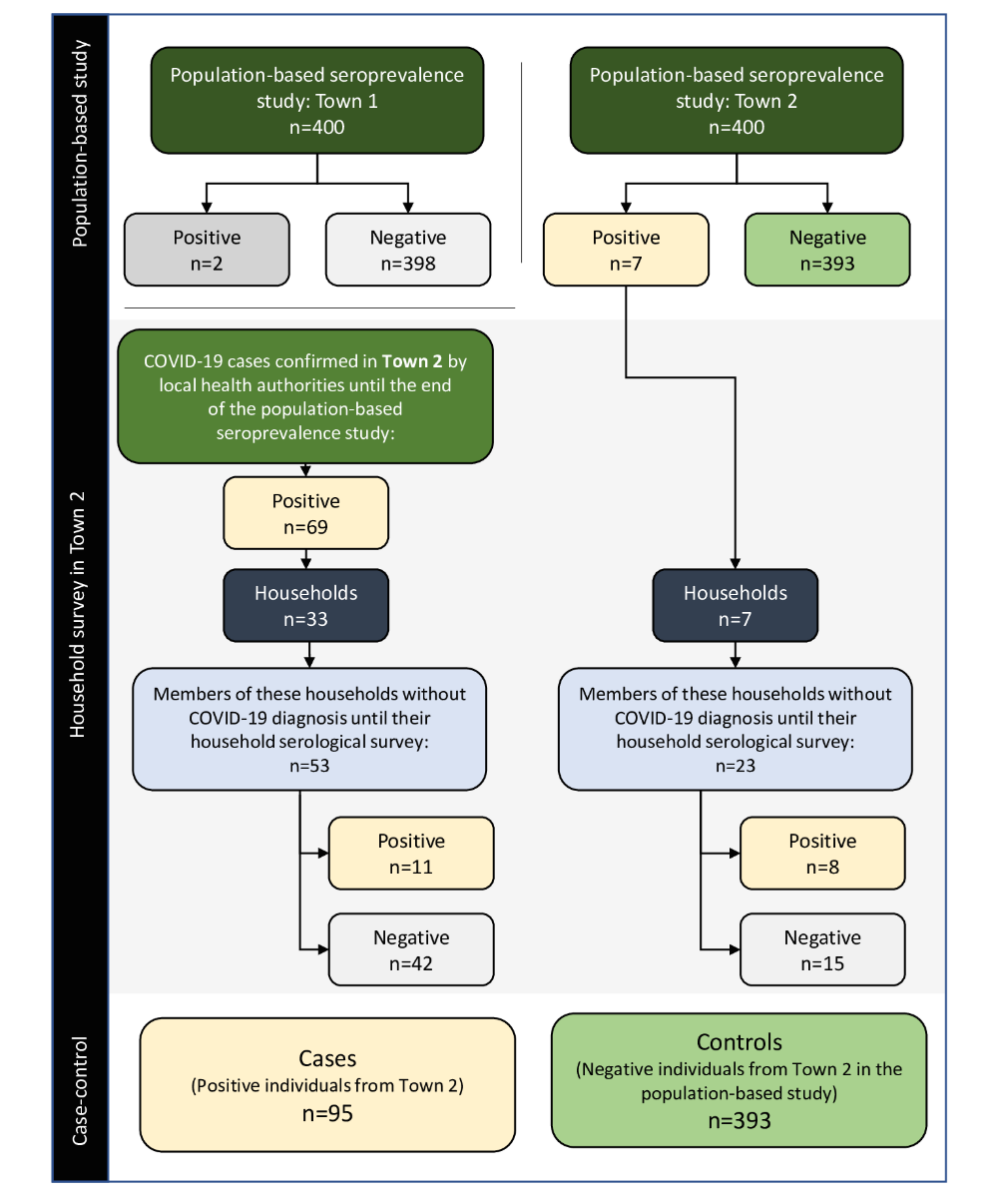
Study design of our population-based, household survey, and case-control study.

At the end, a case-control study was carried out with the information obtained from all participants in Town 2 to identify factors associated with COVID-19 diagnosis. In this study, all individuals with positive testing for COVID-19, identified during the cross-sectional seroprevalence study or by the household survey, were considered cases. All individuals with negative COVID-19 testing during the cross-sectional population-based study were considered as controls (Figure 1).

### Sampling

The sample size for the cross-sectional population-based seroprevalence study was estimated using the online software OpenEpi (OpenEpi Project version 3.01) considering the total population of the urban area, a seroprevalence of 2% [5], and an absolute precision of 1.5%, resulting in a sample of approximately 400 participants in each town. Using maps and census data made available by the Brazilian Institute of Geography and Statistics [6], the total sample (n=400) from each town was divided between all urban census sectors proportionally to their population. Corners of these census sectors were randomly selected for the initial visit, from which a random route was established inside the sector. After the interview and blood collection in the first residence, 4 houses were skipped and a new interview was conducted in the fifth house (commercial properties such as stores, banks, and hotels were not considered), and in cases of refusal, the immediate next house was selected. In the houses participating in this population-based survey, only the resident with the closest birthday was interviewed and serologically tested for COVID-19, and a previous COVID-19 diagnosis was not an exclusion criteria.

For the case-control study, considering the 95 cases and 393 controls, a type I error (α) of .05, and a frequency of exposure of 1% in controls and 8% in cases, the test power (1-β) was between 90% and 95%.

### Study Area

Town 2 was chosen because it is a small town (*small* defined as total population less than 30,000 people) within a radius of 100 km from Town 1 that had the highest number of COVID-19–confirmed cases at the time. Both towns are located in the state of Minas Gerais, in southeastern Brazil, and they have a total population of 25,733 and 17,048 inhabitants, respectively. About 19,004 inhabitants live in the urban area of Town 1 and 11,739 in the urban area of Town 2 [6]. Their current estimated annual gross domestic product (GDP) per capita is US $4706 and US $5497, respectively, and the economy of both is similar, with the service (tertiary) sector comprising nearly 70% of their GDP, while the remaining percentage is mostly represented by coffee- and tomato-related agro-industrial activities [7].

### Serological Testing of COVID-19

At the beginning of each visit, a sample of the participant’s peripheral blood (3 mL) was collected by puncture of the brachiocephalic vein by a trained nurse and then transferred to a serum-separating tube. The tube was stored between 2 °C to 8 °C and transported within 2 hours to the public laboratory of the town Department of Health, where it was immediately centrifuged (2000xg for 10 minutes) and the separate serum was tested for SARS-CoV-2 antibodies using a lateral flow immunoassay according to the manufacturer’s instructions (Hightop SARS-CoV-2 IgM/IgG Antibody Test, Qingdao Hightop Biotech Co, Ltd, China). The sample was considered positive if IgM or IgG antibodies were detectable. The Hightop kit was chosen because robust performance studies [8-11] were available showing that this kit has specificity of 100% for both IgM and IgG, without cross-reactivity even for human seasonal coronaviruses, and an IgG sensitivity of approximately 95% 20 days after the onset of symptoms.

### Collection of Data

The interviews of the population-based seroprevalence study were conducted verbally just after the blood collection, and responses were saved on portable electronic devices using KoBoToolbox software (Harvard University). All interviewers were third- to sixth-year medical students who were centrally trained and were provided with personal protective equipment for each interview. The interviews sought information on the following: sociodemographic and economic characteristics; behavioral variables related to COVID-19; current and previous symptoms compatible with COVID-19; and general health condition, use of medications, and presence of comorbidities. COVID-19 prevention questions were not based on actual efficacious prevention but rather assessed what methods (regardless of effectiveness) people were using.

In Town 2, the subsequent household survey of all positive individuals of the town included serological testing of all household members without previous COVID-19 diagnosis and individual interviews with all household members using the same questionnaire from the population-based study (interview was not duplicated if the participant had already been interviewed during the population-based study). If the household still had members on quarantine or isolation at the date of the survey, the interview and serological test of all household members were postponed to 1 day after the end of that period.

### Definitions: COVID-19 Confirmed Case, Index Case, Logistics Worker, and High-risk Group

For the household survey, the local health authorities gave us a list of all 69 COVID-19 cases confirmed in Town 2 until June 28, 2020, apart from those cases detected by our population-based seroprevalence study. Their definition of confirmed cases included patients with a positive SARS-CoV-2 reverse transcriptase–polymerase chain reaction (RT-PCR) or serology test (IgM or IgG).

The index patient was defined as the most likely first infected member of a household, and it was usually the household member who first presented COVID-19–compatible symptoms. The presence of patients with asymptomatic COVID-19 in the house, the type (RT-PCR or serology) and date of the first positive test of each patient, the history of previous symptoms compatible with COVID-19 (detailed history was particularly important for cases whose COVID-19 diagnosis was based on serology), and the contact tracing information of each household provided by the town Department of Health were also considered during definition of the index patient. For each household, the joint interview conducted with all of its members was also of pivotal importance for defining the index patient and the most likely source of infection to the household. The index date of each household was defined as the date of symptom onset for the index patient or as the date of the first COVID-19–positive test in cases of an asymptomatic index patient. All this information was also used to retrospectively create the probable SARS-CoV-2 transmission chain between different households.

For the purposes of this study, a logistics worker was defined as an individual with an occupation focused on the transportation of people or goods and whose job involves traveling outside the town of residence at least once a week.

An individual was considered as part of the COVID-19 high-risk group if they reported at least one of the following conditions: 60 years or older; chronic obstructive pulmonary disease; pulmonary fibrosis; asthma; heart failure, previous myocardial infarction, atrial fibrillation, coronary artery disease, or other severe heart disease; previous stroke; type 1 or type 2 diabetes mellitus; chronic kidney disease on dialysis or with glomerular filtration rate <60 mL/min; severe liver disease; severe neurologic conditions; chromosomal abnormalities; sickle cell anemia; HIV with a low CD4 cell count or not on HIV treatment; immunocompromised state from blood, bone marrow, or organ transplant; prolonged use of corticosteroids or other immunosuppressant drug; current cancer; current smoker; or BMI of 35 kg/m^2^ or higher.

### Statistical Analysis

The data analysis was performed using STATA software version 14.0 (StataCorp). To calculate the household secondary infection rate, the number of household members with confirmed COVID-19 was divided by the total number of household members excluding the index case. The 95% CIs around the prevalence and secondary infection rates were calculated using the Wilson method.

To investigate the factors associated with the risk of catching COVID-19, case and controls were compared. A logistic regression model was used to evaluate the association between the dependent and independent variables. Occupation was categorized based on the level of essentiality of each occupation during the social restrictions and lockdown measures that had been implemented worldwide and in Brazil at the moment. Univariate analysis was performed for all variables collected, and those with a *P*<.25 were included in the initial multivariate model. The backward method [12] was subsequently adopted and only variables with *P*<.05 remained in the final multivariate model. Among variables that showed collinearity, only the one that was the best predictor (higher log likelihood) was retained.

### Ethical Approval

The study was approved by the research ethics committee of Federal University of Ouro Preto, Brazil (protocol identification number: CAAE - 32267920.7.0000.5150). Informed consent was read and signed by all participants. In case of minors younger than 18 years, written consent was obtained from parents or legal guardians. Literate children and adolescents were also asked to read and sign an assent form.

## Results

### Characteristics of the Population of Town 1 and Town 2

The cross-sectional population-based study gave information about general characteristics of inhabitants from both towns (Table 1). The inhabitants in Town 1 had an average age of 47.2 (SD 20.3) years. The average number of people per household was 3.2 (SD 1.4), and the average number of rooms and bathrooms per house was 6.4 (SD 2.0) and 1.5 (SD 0.9), respectively. Thus, the average number of people per room in each housing unit was 0.5 (SD 0.3). It was also observed that in 38.7% of households there was at least one person 60 years or older.

In Town 2, inhabitants had an average age of 43.5 (SD 21.1) years. The average number of residents per household was 3.2 (SD 1.6), and the average number of rooms and bathrooms was 7.4 (SD 2.2) and 1.5 (SD 0.7), respectively. Thus, the average number of people per room in each housing unit was 0.4 (SD 0.2). In 36.5% of households, there was at least one person 60 years or older.

Other characteristics of the study participants from both towns are detailed in Table 1. Although there are minor differences in socioeconomic and demographic variables between both towns, their preventive behaviors for COVID-19 are similar.

**Table 1:** Cross-sectional population-based study: sociodemographic and behavioral characteristics of Town 1 (n=400) and Town 2 (n=400).

Variable		Town 1, n (%)	Town 2, n (%)	*P* value	
**Sex**					.01
	Male	156 (39.0)	196 (49.0)		
	Female	244 (61.0)	204 (51.0)		
**Race**					.04
	White	211 (52.8)	235 (58.7)		
	Brown-skinned	112 (28.0)	117 (29.2)		
	Black	70 (17.5)	40 (10.0)		
	Other	7 (1.7)	8 (2.1)		
**Age group (years)**					.29
	0-12	23 (5.8)	33 (8.2)		
	13-18	17 (4.2)	22 (5.5)		
	19-30	53 (13.2)	66 (16.5)		
	31-45	81 (20.2)	78 (19.5)		
	46-59	113 (28.3)	109 (27.2)		
	≥60	113 (28.3)	92 (23.0)		
**Occupation**					.01
	Homemaker/unemployed	69 (17.2)	49 (12.2)		
	Retiree	84 (21.0)	54 (13.5)		
	Student/teacher/professor	58 (14.5)	66 (16.5)		
	Rural worker	28 (7.0)	23 (5.8)		
	Storekeeper/clerk/local employee/independent worker	130 (32.5)	155 (38.7)		
	Health care professional	15 (3.8)	13 (3.3)		
	Logistics worker	16 (4.0)	40 (10.0)		
**Smoking status**					.36
	Nonsmoker	252 (63.0)	271 (67.7)		
	Former smoker	79 (19.7)	68 (17.0)		
	Current smoker	69 (17.3)	61 (15.3)		
**BCG^a^ vaccinated?**					.41
	Yes	355 (88.7)	362 (90.5)		
	No	45 (11.3)	38 (9.5)		
**COVID-19 high-risk group?**					.04
	Yes	220 (55.0)	191 (47.8)		
	No	180 (45.0)	209 (52.2)		
**Did you meet someone exclusively for leisure/socializing purposes during the past 10 days?**					.01
	Yes	156 (39.0)	202 (50.5)		
	No	244 (61.0)	198 (49.5)		
**Do you wear mask at work?**					.07
	Yes, all the time	138 (34.6)	131 (32.7)		
	Yes, most of the time	49 (12.2)	33 (8.2)		
	Yes, only sometimes	25 (6.2)	15 (3.8)		
	No	29 (7.2)	34 (8.5)		
	Not applicable (do not work or home office)	159 (39.8)	187 (46.8)		
**Do you wear mask while walking on the streets?**					.24
	Yes, all the time	260 (65.0)	278 (69.5)		
	Yes, most of the time	60 (15.0)	41 (10.3)		
	Yes, only sometimes	34 (8.5)	37 (9.2)		
	No	25 (6.3)	19 (4.7)		
	Not applicable (do not leave the house)	21 (5.2)	25 (6.3)		
**Do you pull the mask down to talk to someone?**					.38
	Yes, always	32 (8.0)	26 (6.5)		
	Yes, sometimes	42 (10.5)	53 (13.3)		
	No	326 (81.5)	321 (80.2)		
**Are you regularly taking exclusively for COVID-19 prevention?**					
	Vitamin or mineral	23 (5.8)	30 (7.5)	.32	
	Hydroxychloroquine	0 (0)	1 (0.2)	.32	
	Herbal medicine	7 (1.8)	2 (0.5)	.09	
	Ivermectin	—^b^	23 (5.8)	N/A^c^	

^a^BCG: Bacillus Calmette Guérin.

^b^Prophylactic use of ivermectin was not assessed in Town 1.

^c^N/A: not applicable.

### Population-Based Seroprevalence and Underreporting

The prevalence of SARS-CoV-2 infection in Town 1 was 0.5% (95% CI 0.13%-1.80%), since two positive cases were found among the 400 participants evaluated in the population-based serological survey. Based on an urban population of 19,004 inhabitants, this prevalence estimate represents 95 people infected, which is 48-fold more than the number of confirmed cases at that moment (although there were no confirmed cases in the town while the study was being planned, 2 cases were reported by local health authorities just before the execution phase of our study). All 2 cases found in the seroprevalence survey and all other 2 cases already reported in the town were logistics workers or their household members.

In Town 2, a total of 7 positive cases were found among the 400 participants in the population-based seroprevalence study, which corresponds to a prevalence of 1.75% (95% CI 0.85-3.57). As the urban population is 11,739 inhabitants, this prevalence estimate represents 205 people infected, which is 3-fold more than the number of confirmed cases (69 cases had already been reported in the town). Most of the cases identified in the seroprevalence survey (4/7, 57.1%) were logistics workers or their household members. None of the cases found in the population-based survey had been previously detected by local health authorities.

### Town 2 Household Survey of COVID-19 Cases

During the household survey of all COVID-19 cases from Town 2, a total of 40 residences were evaluated, 7 of which were residences of COVID-19 cases identified in our population-based seroprevalence study and 33 residences from COVID-19 cases independently confirmed by local health authorities. In these 40 households, there were 152 individuals in total, and the average number of residents per house was 3.8 (SD 1.5). Until the serological survey of all members of these 40 households, the average number of confirmed COVID-19 cases per house was 1.9 (SD 1.4), and this average increased to 2.4 (SD 1.5) after the serological survey (Figure 1). In 70% (28/40) of households, the index case was a logistics worker, and there was at least one logistics worker in 77.5% (31/40) of the households (Table 2).

**Table 2:** Characteristics of all households from Town 2 with at least one COVID-19 case (n=40).

Variable			Households, n (%)
**Did this household receive visitors in the 14-day period before the household index date?**			
	Yes	14 (35)	
	No	26 (65)	
**Was any celebration (eg, barbecue or dinner party) held in this household in the 14-day period before the household index date?**			
	Yes	4 (10)	
	No	36 (90)	
**Did the index case of this household attend any celebration in the 14-day period before the household index date?**			
	Yes	9 (22.5)	
	No	31 (77.5)	
**Is the index case a logistics worker?**			
	Yes	28 (70)	
	No	12 (30)	
**Is any member of this household a logistics worker?**			
	Yes	31 (78)	
	No	9 (23)	
**Measures taken to reduce viral transmission**			
	Sharing the same bed with the infected person was avoided	19 (48)	
	Sharing the same couch with the infected person was avoided	17 (42.5)	
	Sharing eating utensils with the infected person was avoided	16 (40)	
	The infected person washed his own sheets and other bedding	10 (25)	
	The infected person stayed in a separate room, walking out only when absolutely necessary	8 (20)	
	The infected person used a separate bathroom	6 (15)	
**How often did the household members perform hand hygiene while there was one active COVID-19 case in the house?**			
	Frequently	31 (78)	
	Sometimes	6 (15)	
	Rarely	3 (8)	
**Did the active COVID-19 case use to wear mask while near other people in shared areas of the house?**			
	No	27 (68)	
	Yes, always or almost always	12 (30)	
	Yes, only sometimes	1 (3)	

### Household Secondary Infection Rate and Contact Tracing

In Town 2, the secondary positive COVID-19 cases reported that symptoms started on average 5.7 (SD 3.2) days after the index date, with a minimum of 1 and a maximum of 12 days. Among the 112 individuals who lived with the index cases in the 40 households, 55 were also identified as confirmed COVID-19 cases until the end of our study, thus the secondary household infection rate in Town 2 was 49.1% (55/112, 95% CI 40.0-58.7).

**Figure 2:**
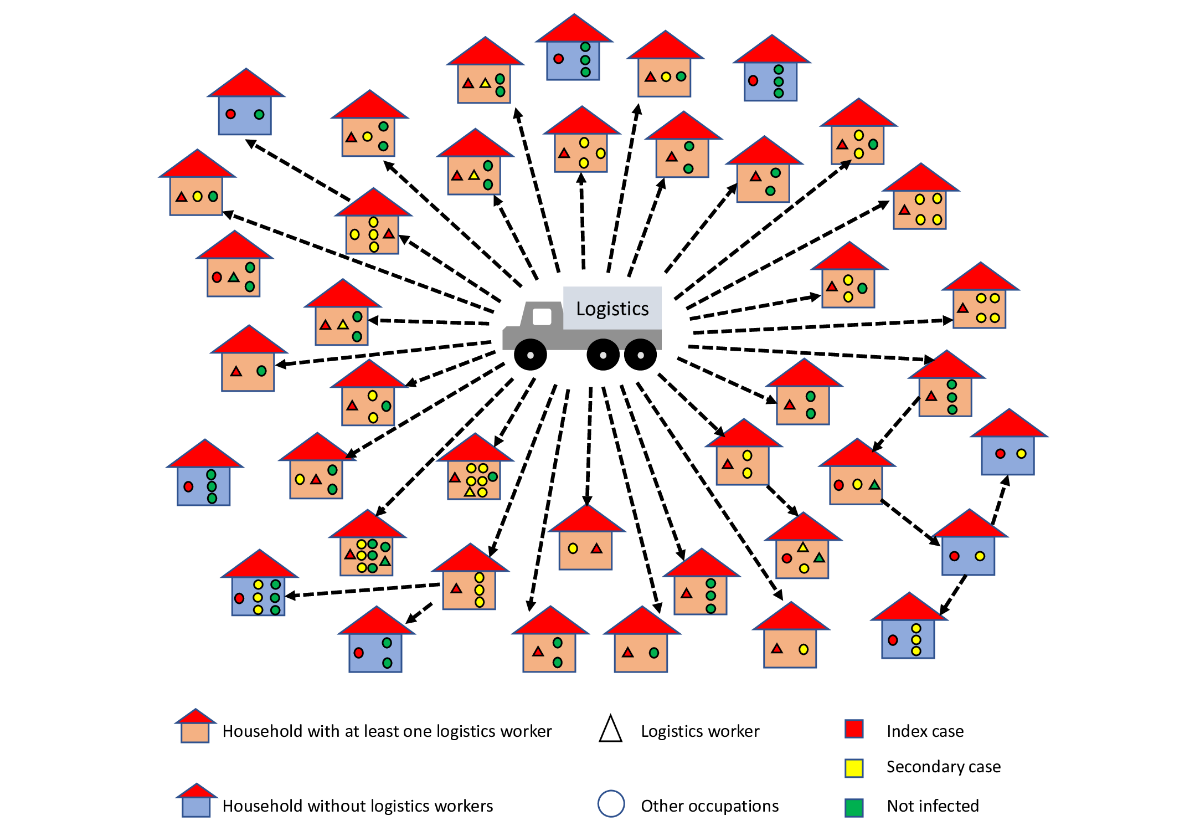
SARS-CoV-2 transmission chains in the first 40 households of Town 2 with COVID-19–confirmed cases. The arrows represent the probable transmission chains.

Using the information about contact tracing provided by local health authorities and the information collected by our interviewers, we retrospectively constructed the probable SARS-CoV-2 transmission chain in all 40 households evaluated in our study from Town 2. It is possible to observe that, in most houses, the index case was a logistics worker (28/40, 70%). In addition, it was noted that the transmission route usually started at homes with logistics workers (Figure 2).

### Factors Associated With Diagnosis of COVID-19

A comparison between cases (n=95) and controls (n=393) was performed by multivariate analysis using the variables obtained from the interviews. The results of the preliminary selection of the variables during univariate analysis (*P*<.25) are shown in Table 3. The variables retained in the final multivariate model (*P*<.05) were to be a logistics worker (odds ratio [OR] 18.0, 95% CI 8.4-38.7) or to live with a logistics worker (OR 6.9, 95% CI 3.3-14.5), to have close contact with a confirmed COVID-19 case (OR 13.4, 6.6-27.3), to live with four or more people (OR 2.7, 95% CI 1.4-5.4), and to be a current smoker (OR 0.2, 0.1-0.7; Table 4).

**Table 3:** Univariate analysis in the case-control study: distribution of COVID-19 cases (n=95) and controls (n=393) from Town 2 according to sociodemographic and behavioral characteristics.

Variables			Case, n (%)		Control, n (%)		Odds ratio (95% CI)		*P* value
**Occupation**									
	Homemaker/unemployed	7 (7.4)		49 (12.0)		1.0 (reference)		N/A^a^	
	Retiree	7 (7.4)		55 (14.0)		0.9 (0.3-2.6)		.78	
	Student/teacher/professor	24 (25.3)		64 (16.3)		2.5 (1.0-6.3)		.05	
	Rural worker	0 (0)		23 (5.8)		N/A		N/A	
	Storekeeper/clerk/local employee/independent worker	19 (20.0)		153 (38.9)		0.8 (0.3-2.1)		.70	
	Health care professional	5 (5.3)		13 (3.3)		2.6 (0.7-9.5)		.15	
	Logistics worker	33 (34.7)		38 (9.7)		5.8 (2.3-14.6)		.01	
**How many people do you live with?**									
	Alone or with 1 person	11 (11.6)		122 (31.0)		1.0 (reference)		N/A	
	With 2 or 3 people	54 (56.8)		220 (56.0)		2.7 (1.4-5.4)		.01	
	With 4 or more people	30 (31.6)		51 (13.0)		6.5 (3.0-14.0)		.01	
**Number of rooms per person in the house**									
	>2	38 (40.0)		225 (57.3)		1.0 (reference)		N/A	
	≤2	57 (60.0)		168 (42.7)		2.0 (1.3-3.2)		.01	
**Do you live with or are you a logistics worker?**									
	No	17 (17.9)		305 (77.6)		1.0 (reference)		N/A	
	Yes, I live with a logistics worker	45 (47.4)		50 (12.7)		16.1 (8.6-30.4)		.01	
	Yes, I am a logistics worker	33 (34.7)		38 (9.7)		15.6 (7.9-30.6)		.01	
**Smoking status**									
	Nonsmoker	73 (76.8)		266 (67.7)		1.0 (reference)		N/A	
	Former smoker	16 (16.8)		66 (16.8)		0.9 (0.5-1.6)		.67	
	Current smoker	6 (6.3)		61 (15.5)		0.4 (0.1-0.9)		.02	
**Did you have close contact^b^ with a COVID-19 case?**									
	No	46 (48.4)		369 (93.9)		1.0 (reference)		N/A	
	Yes	49 (51.6)		24 (6.1)		16.4 (9.2-29.1)		.01	
**Do you have frequent contact^c^ with a logistics worker?**									
	No	33 (34.7)		285 (66.7)		1.0 (reference)		N/A	
	Yes	62 (65.3)		108 (33.3)		5.0 (3.1-8.0)		.01	

^a^N/A: not applicable.

^b^Close contact was defined as being within 6 feet of a person who is infected for at least 15 minutes during a period starting from 2 days before illness onset until the end of isolation of the patient who is infected.

^c^Frequent contact was defined as having close contact at least once a week.

**Table 4:** Multivariate analysis of sociodemographic and behavioral factors associated with COVID-19 diagnosis: case-control study in Town 2.

Variables			Crude odds ratio (95% CI)		Adjusted odds ratio (95% CI)		*P* value
**Do you live with or are you a logistics worker?**							
	No	1 (reference)		1 (reference)		N/A^a^	
	Yes, I live with a logistics worker	16.1 (8.6-30.4)		6.9 (3.3-14.5)		.01	
	Yes, I am a logistics worker	15.6 (7.9-30.6)		18.0 (8.4-38.7)		.01	
**Did you have close contact^b^ with a COVID-19 case?**							
	No	1 (reference)		1 (reference)		N/A	
	Yes	16.4 (9.2-29.1)		13.4 (6.6-27.3)		.01	
**How many people do you live with?**							
	Alone or with 1 person	1 (reference)		1 (reference)		N/A	
	With 4 or more people	6.5 (3.0-14.0)		2.7 (1.1-7.1)		.04	
**Smoking status**							
	Nonsmoker	1 (reference)		1 (reference)		N/A	
	Current smoker	0.4 (0.1-0.9)		0.2 (0.1-0.7)		.01	

^a^N/A: not applicable.

^b^Close contact was defined as being within 6 feet of a person who is infected for at least 15 minutes during a period starting from 2 days before illness onset until the end of isolation of the patient who is infected.

## Discussion

### Principal Findings

This study started as a seroprevalence survey in Town 1 (Brazil) to verify if SARS-CoV-2 had already spread even in Brazilian small towns without COVID-19–confirmed cases. The survey showed a low seroprevalence in that town (0.5%), but the identified cases shared a common denominator: they were all related to logistics workers. To confirm the influence of these workers in the spread of the virus in the region, we conducted both a seroprevalence survey and a case-control study in another nearby small town (Town 2) that already had a higher number of confirmed cases. This new survey showed a seroprevalence of 1.75% in the town, and 57.1% of the survey-identified cases were directly related to logistics workers. The case-control study showed that the occupation with the highest risk for COVID-19 are the ones related to logistics (risk higher than health care occupations) and that living with a logistics worker put inhabitants of the town at a high risk of acquiring COVID-19. In addition, we showed that the chain of transmission usually starts in households with logistics workers.

Our study took place between May and the end of June 2020, a time period when the virus was moving from bigger Brazilian cities and capitals toward small towns and rural areas [5]. This move was slow probably because, since April 2020, most cities in Brazil, including the two towns of this study, had already implemented many social contact restrictions and laws to mandate wearing a face mask [5,13]. So our study captures a screenshot of factors that allowed the expansion of the pandemic to low densely populated areas even in a scenario of gathering restrictions, face mask mandates, and other lockdown measures.

Throughout this pandemic, logistics workers kept *on the road* were of vital importance to maintain a continued supply of essential goods to allow people to *stay at home*. Besides that, medical supply chains are reliant on truck drivers and other logistics workers, and will continue to be as treatments and vaccines are approved, manufactured, and distributed [14]. As logistics workers strive to meet the unprecedented demands due to the current pandemic, their movement patterns and social interactions are unique and of foremost epidemiological significance [14,15].

As our study captures the moment of initial local viral transmission (as reflected by low seroprevalence in both towns), our results portray what triggers community transmission (ie, what allows transitioning from imported cases to community transmission). The identification of these triggers is important for slowing down the spread of a pathogen and is, thus, a strategy for public health security. Although imported cases are easier to manage, community transmissions are hard to trace, can grow quickly, and easily threaten local public health systems [16].

Logistics workers have been shown to spread infectious diseases such as HIV and syphilis across geographic lines, both locally and in distant areas [17]. The same seems to be true regarding SARS-CoV-2, and in fact, the transport sector was substantially hit during this pandemic to slow the spread of the virus. This hit on transportation was mainly focused on international travel, air transport, and tourism [18,19] while undermining the importance of essential local logistics workers. This underestimation is reflected by the lack of COVID-19 public health strategies and research studies focused in this group.

Our study shows that, in a scenario of lockdown and mask mandates in small Brazilian towns, essential local logistics workers and their household members had the highest risk of contracting COVID-19 during the initial phase of the local epidemic. Thus, these workers are an important core group that spreads the infection to the general population, allowing the initiation of community transmissions. Noteworthy, all cases found in Town 1 during our study were likely imported cases, and at the time, there was no evidence of community transmission in that town.

Our study is among the first to show the peculiar role of these workers in the spread of SARS-CoV-2. To our knowledge, only one study from Uganda has pointed it out so far. The authors [20] reviewed the first 10 weeks of press releases from the Uganda Ministry of Health from the day when the first case was announced. At the end of these 10 weeks, 442 COVID-19 cases had been confirmed, most of which (71.8%) were truck drivers. Besides that, the majority of community cases identified have had contact with these drivers. They concluded that the epidemic in Uganda, a country that was in national lockdown during those 10 initial weeks, was literally being driven by truck drivers.

Considering our findings, one factor that probably explains why Town 2 had a seroprevalence almost four times higher than Town 1 is the fact that the population of Town 2 had a significantly higher proportion of logistics workers (Table 1). Another factor is that the study was done in Town 2 later than in Town 1, but this time gap alone likely does not explain the difference between the two towns because data from nearby cities indicate that seroprevalence in the area was stable and did not even double during this interval [5,21]. Apart from that, it should be observed that both towns have similar economies, are close to each other within the same state, and were taking the same lockdown measures in accordance with guidelines from their State Health Department. In addition, the population-based survey showed similar compliance with wearing a mask in both towns, with nearly all participants wearing masks when going out, which is similar to attitudes and practices toward COVID-19 in other countries at that time [22,23].

Our multivariate model showed that the risk of getting COVID-19 is almost three times higher in individuals who share their household with four or more members, compared to those who live alone or with only one person. We also found a household secondary infection rate of 49.1% (55/112), suggesting a high rate of intrafamily transmission. This rate is similar to rates found by other studies in western countries, such as 53% in the United States [24] and 43% in Italy [25], but it is higher than rates found in eastern countries, such as 30% in China [26] and 11.8% in South Korea [27], probably due to different culture and customs inside the household environment. For example, although one Chinese study [28] found that 93.5% of patients isolated at home with COVID-19 were fully compliant to wearing masks during family activities in shared areas of the house; only in 30% of households from our study did the confirmed case wear a mask in the same circumstance.

One variable that in our multivariate analysis was associated with lower risk of getting COVID-19 is smoking. The protective effect of smoking in COVID-19 has been a consistent finding across many published studies [29,30], but it should be viewed with caution because this protective effect is unlikely to outweigh the numerous proven adverse health effects of smoking. Besides that, although smokers may have a reduced chance of getting COVID-19, they have a higher risk of severe disease in case they are infected [31].

Our findings in this study are subject to a number of limitations. First, as our research was conducted only in two towns from the southern region of the state of Minas Gerais, our results may not be generalizable to other Brazilian states and even to other regions of the state of Minas Gerais. Second, regarding the calculated household secondary infection rate, although living in the same household might convey a high risk of acquiring infection, some infections might have originated outside the household, leading to a higher apparent secondary infection rate. Third, even though not all patients infected with SARS-CoV-2 will become IgM or IgG positive [32], we chose an antibody test kit that has a high sensitivity validated by many robust performance studies [8-11]. In addition, as our study is focused on the initial phase of local transmission, our results are unlikely to be affected by the fact that SARS-CoV-2 antibodies may become undetected in some individuals 3 months after recovery [33]. Fourth, recall bias and selection bias are intrinsic limitations of case-control studies. Recall bias was probably minimal in our study because we investigated variables that are easy to recollect (eg, occupation, COVID-19–related behavior, and contact tracing) especially because they are mostly related to a pandemic that impacted everyone’s life, particularly in a small town with many implemented social contact restrictions and lockdown measures. Selection bias related to controls was probably minimal too because controls came from the initial population-based survey, so the control group is representative of the local population. In addition, we had more than 4 controls for each case, which is an optimal ratio to increase statistical power of a case-control study [34].

When it comes to cases, a source of selection bias is the fact that our case-control study also includes cases from Town 2 that were identified by local health authorities, and there may be biases in how they identified the cases. It is important to note that Brazil has a free and universal public health system and that COVID-19 testing in Brazil was scarce at the moment [35,36], so health authorities in Town 2 were testing only patients who were symptomatic, but no age or occupation was being prioritized. Under this scenario, it is not likely that our cases are biased toward a community segment, but they may be biased toward a group more likely to become symptomatic. Nevertheless, we think the probability of this bias in our main findings is low because, as already mentioned, the majority of cases detected in the population-based seroprevalence survey (which included participants regardless of symptoms) were also cases related to logistics workers.

Recent data from the Brazilian Ministry of Economy [37] has shown that truck drivers and bus drivers were the two occupations with the highest increase in job termination due to death (both with 407% increases) when compared with the first bimester of 2019 and 2021, which is three times higher than health care occupations. This data reinforces the validity of our results and indicates the need to focus on logistics workers to determine COVID-19 public policies, including prevention of the spread of novel variants.

### Conclusions

Our study shows a strong association between being a logistics worker and the risk of SARS-CoV-2 infection, and points out the role of these workers as a core group that brings the virus to Brazilian small towns. These findings indicate the need to focus on these workers to determine COVID-19 prevention and control strategies, as they are important triggers for initiation of local community transmission and may be triggers for the spread of novel concerning variants in areas already under control. In light of this evidence, logistics workers should also be prioritized for SARS-CoV-2 vaccination and sentinel genomic surveillance, especially in areas similar to those of our study.

## Abbreviations

GDP: gross domestic product
OR: odds ratio
RT-PCR: reverse transcriptase–polymerase chain reaction
